# Records of *Hedotettix* and *Teredorus* in Thailand with the description of three new species (Orthoptera, Tetrigidae)

**DOI:** 10.3897/zookeys.556.6002

**Published:** 2016-01-21

**Authors:** Ling-Sheng Zha, Ting-Chi Wen, Ji-Chuan Kang, Kevin D. Hyde

**Affiliations:** 1 Institute of Excellence in Fungal Research, School of Science, Mae Fah Luang University, Chiang Rai 57100, Thailand; 2 School of Life Sciences, Huaibei Normal University, Huaibei 235000, Anhui, China; 3 Engineering Research Center of Southwest Bio-Pharmaceutical Resources, Ministry of Education, Guizhou University, Guiyang 550025, Guizhou, China

**Keywords:** Tetriginae, taxonomy, pygmy grasshopper, newly recorded genus, biology, Chiang Rai

## Abstract

We are studying the fungi associated with insects in northern Thailand and as a result several rarely collected insect species have been uncovered. The genera *Hedotettix* with one new species and *Teredorus* with two new species are reported from Thailand. *Hedotettix triangularis* Zha & Hyde, **sp. n.**, *Teredorus chiangraiensis* Zha & Hyde, **sp. n.** and *Teredorus combfemorus* Zha & Hyde, **sp. n.** are introduced, described and photographed and compared with other species. Keys to species of *Hedotettix* and *Teredorus* from Thailand are provided.

## Introduction

The genera *Hedotettix* and *Teredorus* belong to Tetriginae, Tetrigidae of Orthoptera. *Hedotettix* includes 46 species (Ou et al. 2014, Zheng 2014a, 2014b, Eades et al. 2015), and has a worldwide distribution; only one species, *Hedotettix gracilis* (De Haan, 1843) is known from Thailand (Ingrisch 2001, Zheng 2005, 2014a). *Teredorus* includes 29 species; although they mainly occur in South America, China, India and Nepal (Deng et al. 2014), there are no records of this genus in Thailand.

The aim of this paper is to report two species of *Hedotettix* and two of *Teredorus* from Thailand. *Hedotettix triangularis* Zha & Hyde, sp. n., *Teredorus chiangraiensis* Zha & Hyde, sp. n. and *Teredorus combfemorus* Zha & Hyde, sp. n. are described and illustrated as new to science and compared with other similar species. Keys to species of both *Hedotettix* and *Teredorus* in Thailand are provided.

## Material and methods

Specimens were examined and photographed using a stereo microscope (Olympus Corporation, SZX16, Tokyo, Japan). Morphological terminology and measurements follow those of Vickery and Kevan (1983) and Zheng (2005). Measurements are given in millimeters (mm). Holotypes are deposited in the herbarium of Mae Fah Luang University (MFLU), Chiang Rai, Thailand, paratypes and research specimens in both MFLU and the Specimen Room of the School of Life Sciences, Huaibei Normal University (HNU), Huaibei, Anhui, China.

## Taxonomy

### Hedotettix

Taxon classificationAnimaliaOrthopteraTetrigidae

Bolivar, 1887 in Thailand

#### Key to species of *Hedotettix* Bolivar, 1887 in Thailand

**Table:** 

1	Anterior margin of pronotum slightly obtusely protruding; apex of posterior angle of lateral lobe of pronotum rounded; hind process reaching middle of hind tibia; upper valvula of female ovipositor narrow and elongate, 3.5 times as long as wide	***Hedotettix gracilis* (De Haan, 1843)**
–	Anterior margin of pronotum truncate; apex of posterior angle of lateral lobe of pronotum acutely angled or very short truncate; hind process reaching one third of hind tibia; upper valvula of female ovipositor broad and short, only 2.5 times as long as wide	***Hedotettix triangularis* sp. n.**

### Hedotettix gracilis

Taxon classificationAnimaliaOrthopteraTetrigidae

(De Haan, 1843)

#### Specimens examined.

1 female (No. 15-0624, MFLU) and 2 female (HNU), Thailand, Chiang Rai, 31 Oct. 2014, collected by ZHA Ling-Sheng.

#### Distribution.

Thailand (Chiang Rai).

### Hedotettix triangularis

Taxon classificationAnimaliaOrthopteraTetrigidae

Zha & Hyde sp. n.

http://zoobank.org/76583B54-E7B9-43D6-8D4E-D8FF4529AA77

Figs 1 , 2

#### Diagnosis.

**Figure 1.**
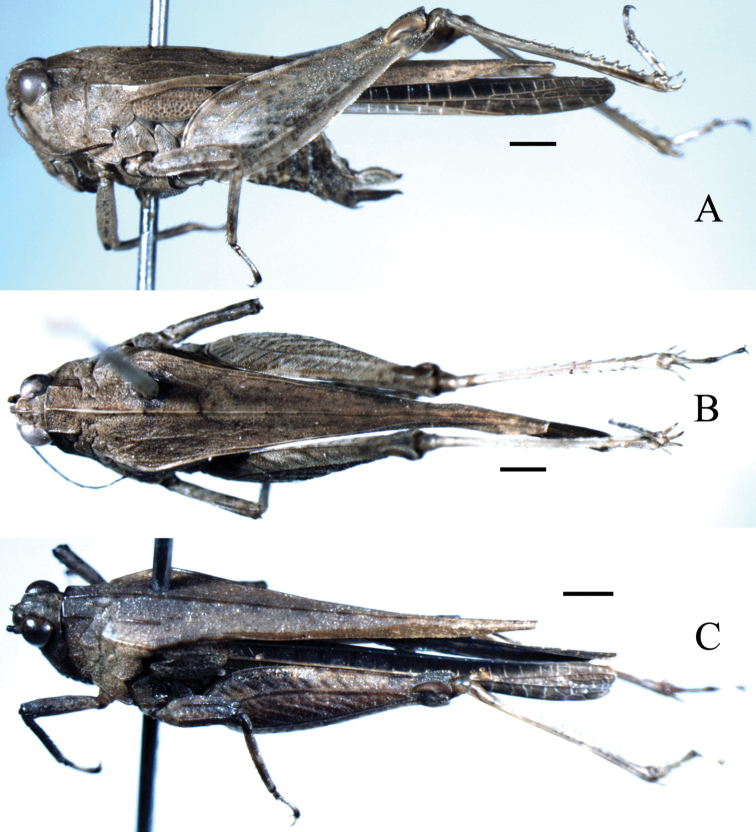
*Hedotettix triangularis* sp. n.: **A** lateral view of female **B** dorsal view of female **C** oblique view of male. Scale bars: 1.0 mm.

**Figure 2.**
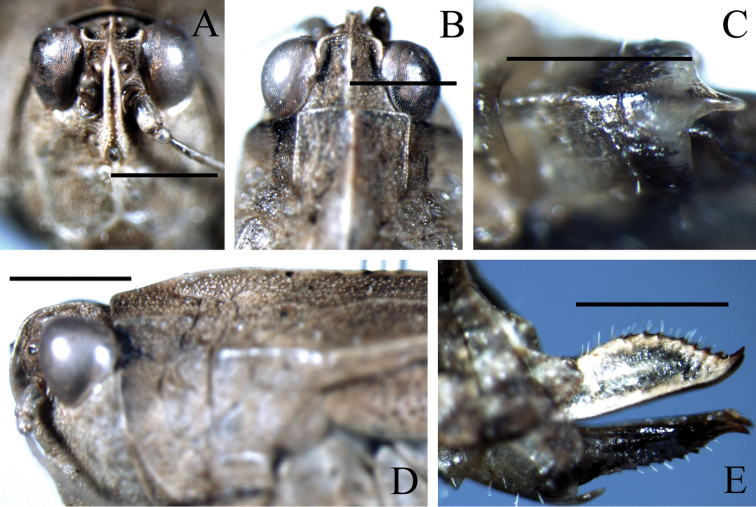
Female of *Hedotettix triangularis* sp. n.: **A** frontal view of head **B** dorsal view of head and anterior pronotum **C** ventral view of subgenital plate **D** lateral view of head and anterior pronotum **E** lateral view of ovipositor. Scale bars: **A–B, D–E**:1.0 mm, **C**: 0.5 mm.

*Hedotettix triangularis* sp. n. is similar to *Hedotettix gracilis* (De Haan, 1843) (Zheng 2005), the former differs from the latter by: 1) anterior margin of pronotum truncate (Fig. 2B); 2) apex of posterior angle of lateral lobe of pronotum acutely angled or very short truncate, not rounded; 3) hind process short, reaching one third of hind tibia (Fig. 1B, C); 4) upper valvula of female ovipositor distinctly short, only 2.5 times as long as wide (Fig. 2E).

#### Description.

Female. Body size medium.

*Head*. Head not protruding over level of pronotal surface, vertex 1.1 times as wide as one eye; anterior margin of vertex arcuate, protruding forward and slightly surpassing beyond anterior margins of eyes, lateral margin distinctly folded upwards; median carina protruding forward and surpassing beyond anterior margin of vertex, conspicuous in anterior half while obscure or disappearing in posterior half, both sides of median carina distinctly concave (Fig. 2B); vertex together with frontal ridge rounded, which is visible before eyes in profile, not concave between lateral ocelli (Fig. 2D); longitudinal furrow decidedly narrower than width of first segment of antenna (0.7–0.8 times), and nearly parallel below level of antennae (Fig. 2A); antenna filiform and short, 16-segmented, length of a segment in middle about 4 times its width, inserted slightly above level of lower margins of eyes (Fig. 2A); eyes globose, lateral ocellus situated slightly above middle of inner margin of eye (Fig. 2A).

*Thorax*. Anterior margin of pronotum truncate, midkeel of pronotum complete and distinct (Figs 1B, 2B); pronotal disc smooth, with numerous fine granules, pronotum slightly tectiform in anterior half and long cone-shaped in posterior half (Fig. 1A, B); in profile upper margin of pronotum arcuate in anterior half (the highest point located between transverse sulcus) while straight in posterior half (Fig. 1A); lateral keels of prozona conspicious and parallel; shoulders broad, then gradually constricted backward; abbreviated carinae elongate or shortened, and present, obscure or absent between shoulders; humeral angle obtusely angled; length of distal part of hind process which surpass beyond apex of hind femur 1.5–2.0 mm, reaching one third of hind tibia (Fig. 1B); posterior angle of lateral lobe of pronotum extending downwards, nearly triangulate, apex acutely angled or very short truncate backwards, posterior margin of each lateral lobe with two concavities; visible part of tegmina ovate, 2.5–2.6 times as long as wide (Fig. 1A); length of distal part of hind wing which surpass beyond apex of hind process of pronotum 1.3–1.8 mm, and reaching about two thirds of hind tibia (Fig. 1A, B); fore femur slender and cylindrical, upper and lower margins straight; upper margin of mid femur slightly undulate, lower margin undulate; middle femur flat, distinctly narrower than width of visible part of tegmen (Fig. 1A); upper and lower margins of mid and hind femora finely dentate, hind femur about 3.0 times as long as wide; antegenicular denticles nearly right angled, genicular denticles fingered extending backward and apex triangulate (Fig. 1A); outer side of hind tibia with 9–11 spines, inner side with 6–9 spines; first hind tarsal segment about 2 times third in length, third pulvillus longer than first and second, apices of first and second pulvilli sharp, apex of third pulvillus nearly right angled.

*Abdomen*. Ovipositor: upper valvula about 2.5 times as long as wide, outer margins of upper and lower valvulae with small saw-like teeth (Fig. 2E); posterior margin of subgenital plate truncate, in the middle acutely triangularly protruding, which is slightly folded inward (Fig. 2C).

*Coloration*. Body yellowish brown. Antenna yellowish brown and distal segments dark brown; hind wings dark brown; for and mid tibiae with 3 obscure dark brown bands (basal and middle bands small while distal band big) respectively; hind tibia light yellowish brown, distal part obscure dark brown.

#### Male.

Body size slightly smaller and slender than female (Fig. 1C). Antenna 15-segmented; middle femur: slightly narrowing from basal to distal side, basal part slightly thicker than distal part, upper margin slightly arcuate and lower margin nearly straight, slightly wider than visible part of tegmen; subgenital plate: cone-shaped, apex notched and not bidentate. Other characters same as female.

#### Measurements.

Length of body ♂7.0–8.0 mm, ♀9.0–10.5mm; length of pronotum ♂9.3–9.8 mm, ♀10.8–11.5 mm; length of hind femur ♂4.5–5.0 mm, ♀5.5–6.0 mm.

#### Type material.

Holotype female (Nos. 15-0625, MFLU), Thailand, Chiang Rai, N20°16'17", E99°48'13", 1076.4 m alt, 30 Sep. 2014, collected by Ling-Sheng ZHA. Paratypes: 2 males and 1 female (Nos. 15-0626, 15-0627, 15-0628, MFLU) and 1 male (HNU), same data as holotype; 2 males and 2 females (HNU), Thailand, Chiang Rai, N20°9'16", E99°37'21", 1504.2 m alt, 22 Oct. 2014, collected by Ling-Sheng ZHA.

#### Biology and ecology.

*Hedotettix triangularis* sp. n. inhabits open meadow in tropical regions. Color of margin of pronotum green when alive which is just like color of the meadow where they inhabit. From collecting time we infer they overwinter as adults.

#### Etymology.

This new species’ name derives from Latin *triangular*, which means posterior angles of lateral lobes of pronotum triangulate.

#### Distribution.

Thailand (Chiang Rai).

### Teredorus

Taxon classificationAnimaliaOrthopteraTetrigidae

Hancock, 1907

#### Type species.

*Teredorus stenofrons* Hancock, 1907, southern America, by original designation.

#### Note.

*Teredorus* Hancock, 1907, is newly recorded genus for Thailand.

#### Key to species of *Teredorus* Hancock, 1907 in Thailand

**Table:** 

1	Lateral keels of prozona normal, slightly shorter than width between them; hind process of pronotum short, never reaching two thirds of hind tibia; lower margins of fore and mid femora normal, not comb-shaped; lower outer side of hind femur distinctly black	***Teredorus chiangraiensis* sp. n.**
–	Lateral keels of prozona short, only 0.5 time as long as width between them; hind process of pronotum elongate, reaching three quarters of hind tibia; lower margins of fore and mid femora comb-shaped; lower outer side of hind femur partially dark brown	***Teredorus combfemorus* sp. n.**

### Teredorus chiangraiensis

Taxon classificationAnimaliaOrthopteraTetrigidae

Zha & Hyde sp. n.

http://zoobank.org/BC3DACC8-22A7-4507-917D-6BA32729B6FF

Figs 3 , 4

#### Diagnosis.

**Figure 3.**
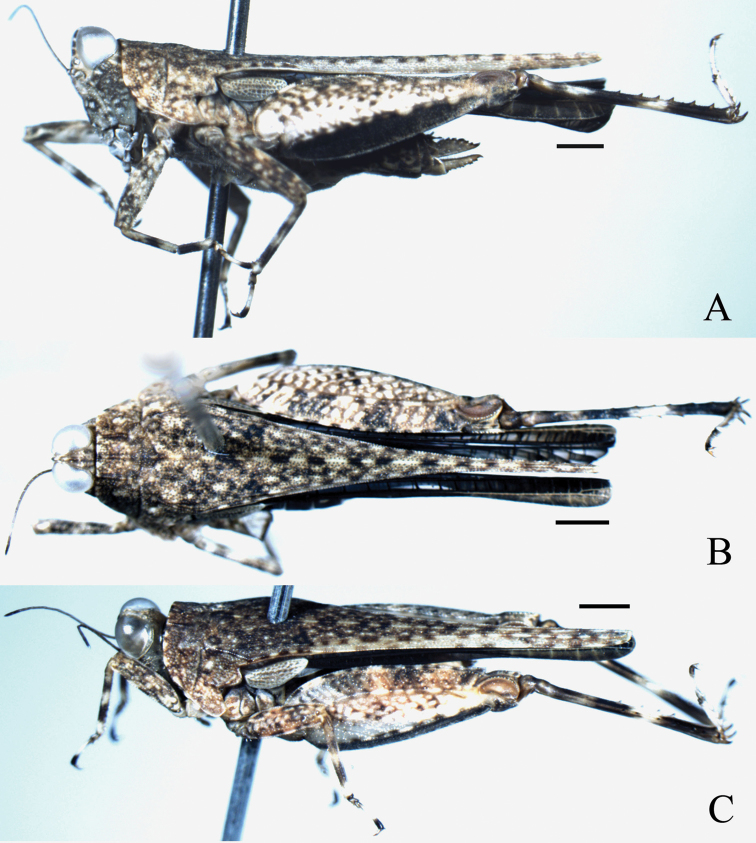
*Teredorus chiangraiensis* sp. n.: **A** lateral view of female **B** dorsal view of female **C** oblique view of male. Scale bars: 1.0 mm.

**Figure 4.**
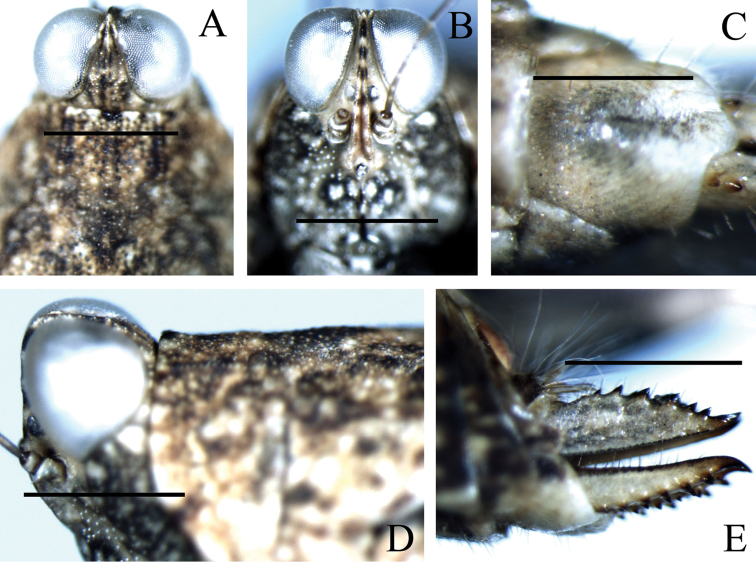
Female of *Teredorus chiangraiensis* sp. n.: **A** dorsal view of head and anterior pronotum **B** frontal view of head **C** ventral view of subgenital plate **D** lateral view of head and anterior pronotum **E** lateral view of ovipositor. Scale bars **A–B, D–E**: 1.0 mm, **C**: 0.5 mm.

*Teredorus chiangraiensis* sp. n. is similar to *Teredorus combfemorus* sp. n., the former differs from the latter mainly by: 1) lateral keels of prozona slightly shorter than width between them (Fig. 4A), while only 0.5 time occurs in the later (Fig. 6D); 2) hind process of pronotum short, never reaching two thirds of hind tibia (Fig. 3A, C); 3) lower margins of fore and mid femora normal, not comb-shaped; 4) lower outer side of hind femur distinctly black (Fig. 3A, C) (also see in Table 1).

**Table 1.** Main differences among *Teredorus chiangraiensis* sp. n., *Teredorus combfemorus* sp. n. and *Teredorus choui* Zheng, Ou & Lin, 2012.

Characters	*Teredorus chiangraiensis*	*Teredorus combfemorus*	*Teredorus choui*
Lateral ocellus situated on inner margin of eye	Lower 1/5	Lower 1/4	Nearly in the middle
Width of longitudinal furrow than width of 1st segment of antenna	Narrower	Equal	Wider
Lateral keel of prozona	Normal (slightly shorter than width between them)	Short, 0.5 time the width between them	Short
Distal part of hind process which surpass apex of hind femur	1.5–2.5 mm	3.3 mm (♀)	♂3.5–4.0 mm, ♀ 2.8–3.0 mm
Apex of posterior angle of lateral lobe of pronotum	Nearly truncate	Nearly truncate	Rounded
Lower margins of fore and mid femora	Normal	Comb-shaped	Normal
Color of lower outer side of hind femur	Black	Partially dark brown	The same color as body
Upper valvulae of female	3.0 times its width	3.0 times its width	2.0 times its width

#### Description.

Female. Body size small and slender, length of body (from vertex to apex of hind process) about 3.3 times its width (between posterior angles of lateral lobes of pronotum) (Fig. 3B).

*Head*. Head distinctly protruding over level of pronotal surface (Fig. 4D); in dorsal view, vertex strongly contracted forward, two eyes nearly connected with each other on anterior margin of vertex, vertex not protruding beyond anterior margins of eyes; lateral margins folded upward and up to same height as anterior part of median carina; median carina conspicuous and protruding in anterior half, while obscure in posterior half (Fig. 4A); vertex a little visible before eyes in lateral view, vertex together with frontal ridge right angled, frontal ridge straight and not concave between lateral ocelli, slightly arc-protruding between antennae (Fig. 4D), longitudinal furrow narrower than first segment of antenna in width; antenna filiform, 16-segmented, inserted below lower margin of eyes (Fig. 4B), mid segment 5–6 times as long as wide; eyes globose, erected above level of pronotal surface, lateral ocellus situated on one fifth of lower inner margin of eye (Fig. 4B, D).

*Thorax*. Disc of pronotum smooth, with numerous small granules, midkeel of pronotum complete; in profile upper margin of pronotum straight, only a little protruding before shoulders (Fig. 3A, B); anterior margin of pronotum truncate, lateral keels of prozona conspicuous and parallel (Fig. 4A), humeral angle obtusely angled, abbreviated carinae absent between shoulders; hind process of pronotum narrow, long cone-shaped, surpassing beyond apex of hind femur and not reaching or slightly surpassing beyond middle of hind tibia (length of distal part which surpass beyond apex of hind femur variable between 1.5 and 2.5 mm, pronotum 4.0–5.7 times as long as the distal part) (Fig. 3B); posterior angle of lateral lobe of pronotum extending downwards, apex nearly truncate, posterior margin of each lateral lobe with two concavities; visible part of tegmina ovate, apex narrowly rounded, 2.8 times as long as wide; hind wings developed, reaching or slightly surpassing beyond apex of hind process of pronotum (Fig. 3A, B); upper and lower margins of all femora finely dentate; upper margin of fore femur slightly arcuate, distal part of lower margin slightly concave; upper margin of mid femur nearly straight, lower margin slightly undulate; mid femur slightly wider than visible part of tegmen; hind femur about 3.1 times as long as wide, antegenicular triangulate, genicular denticles fingered extending backward and apex triangulate; outer side of hind tibia with 6–7 spines, inner side with 4–5 spines; first segment of posterior tarsus equal to third in length, three pulvilli nearly equal in length, apices of all pulvilli obtuse.

*Abdomen*. Ovipositor: upper valvula about 3.0 times as long as wide, outer margins of upper and lower valvulae with small saw-like teeth (Fig. 4E); posterior margin of subgenital plate truncate and in the middle triangularly protruding which is folded inward (Fig. 4C).

*Coloration*. Body dark brown. Antenna brown, colour of distal segments deep; hind wings black; all femora with the same color as body; lower outer side of hind femur black, inner side of hind femur yellowish brown; all tibiae yellowish brown, with 3 black bands respectively (basal band small while middle and distal bands big).

#### Male.

Slightly smaller than female (Fig. 3C). Antenna 15-segmented Hind femur slightly stubby, about 2.8 times as long as wide; subgenital plate briefly cone-shaped, apex notched and not bidentate. Other characters same as female.

#### Measurements.

Length of body (from vertex to apex of abdomen) ♂6.5–7.0 mm, ♀8.0–8.5 mm; length of pronotum ♂8.5–10.0 mm, ♀9.3–10.0 mm; length of hind femur ♂4.7–5.0 mm, ♀5.2–5.5 mm.

#### Type material.

Holotype female (Nos. 15-0629, MFLU), Thailand, Chiang Rai, N20°19'43", E 99°51'49", 404.4 m alt, 25 Nov. 2014, collected by Ling-Sheng ZHA. Paratypes: 2 males and 1 female (Nos. 15-0630, 15-0631, 15-0632, MFLU), 3 males and 2 females (HNU), same data as holotype.

#### Biology and ecology.

*Teredorus chiangraiensis* Zha & Hyde, sp. n. inhabits stony place on border of a stream in tropical region. From collecting time we infer they overwinter as adults.

#### Etymology.

This new species is named after Chiang Rai, its type locality.

#### Distribution.

Thailand (Chiang Rai).

### Teredorus combfemorus

Taxon classificationAnimaliaOrthopteraTetrigidae

Zha & Hyde sp. n.

http://zoobank.org/7E2D87D4-CBBE-47D3-A479-F4F0A6595E8A

Figs 5 , 6

#### Diagnosis.

**Figure 5.**
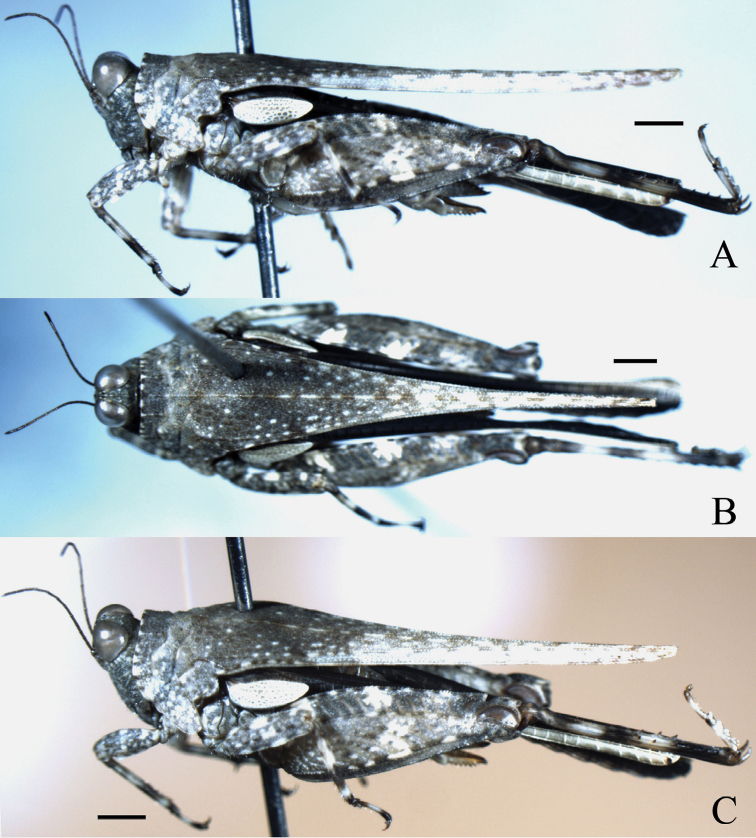
Female of *Teredorus combfemorus* sp. n.: **A** lateral view **B** dorsal view **C** oblique view. Scale bars: 1.0 mm.

**Figure 6.**
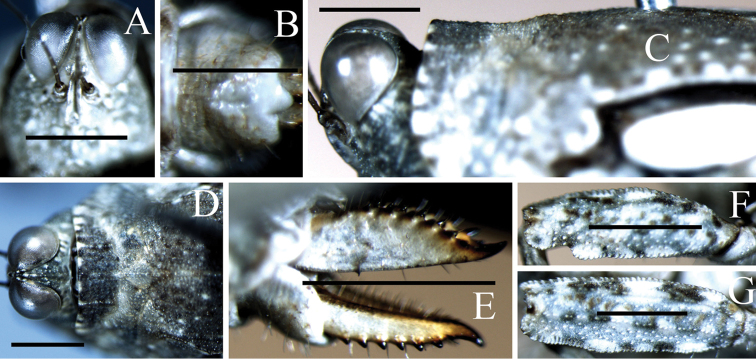
Female of *Teredorus combfemorus* sp. n.: **A** frontal view of head **B** ventral view of subgenital plate **C** lateral view of head and anterior pronotum **D** dorsal view of head and anterior pronotum **E** lateral view of ovipositor **F** lateral view of fore femur **G** lateral view of mid femur. Scale bars **A, C–G**: 1.0 mm, **B**: 0.5 mm.

*Teredorus combfemorus* sp. n. is similar to *Teredorus choui* Zheng, Ou & Lin, 2012, the former differs from the latter mainly by: 1) lateral ocellus situated on a quarter of lower inner margin of eye (Fig. 6A); 2) apex of posterior angle of lateral lobe of pronotum nearly truncate (Fig. 5A, C), not rounded; 3) lower margins of fore and mid femora comb-shaped (Fig. 6F, G); 4) upper valvulae of female 3.0 times as long as wide (Fig. 6E) (also see in Table 1).

#### Description.

Female. Body size small and slender, length of body (from vertex to apex of hind process) 3.5 times its width (between posterior angles of lateral lobes of pronotum) (Fig. 5B).

*Head*. Head distinctly protruding over level of pronotum; in dorsal view, vertex strongly contracted forward and two eyes nearly connected with each other on anterior margin of vertex, vertex not protruding beyond anterior margins of eyes; lateral margins folded upward and up to the same height as anterior median carina; median carina conspicuous and protruding in anterior half, while obscure in posterior half (Fig. 6D); vertex a little visible before eyes in lateral view, vertex together frontal ridge forming right angled, frontal ridge straight and not concave between lateral ocelli, slightly arc-protruding between antennae (Fig. 6C), longitudinal furrow nearly as wide as first segment of antenna; antenna filiform, 15-segmented, inserted below lower margins of eyes (Fig. 6A), mid segment 5–6 times as long as wide; eyes globose, erected above level of pronotal surface, lateral ocellus situated on a quarter of lower inner margin of eye (Fig. 6A, C).

*Thorax*. Disc of pronotum smooth, with numerous small granules, midkeel of pronotum complete; upper margin of pronotum with a very small protrusion before shoulders, in profile upper margin of pronotum slightly undulate (nearly straight) before shoulders and straight behind shoulders (Fig. 5A–C); anterior margin of pronotum truncate, lateral keels of prozona conspicuous and parallel, about 0.5 times as long as the width between them (Fig. 6D); humeral angle obtusely angled, abbreviated carinae absent between shoulders; hind process of pronotum narrow, long cone-shaped, reaching three quarters of hind tibia (length of distal part which surpass beyond apex of hind femur 3.3 mm, pronotum about 3.5 times as long as the distal part) (Fig. 5B); posterior angle of lateral lobe of pronotum extending downwards, apex nearly truncate, posterior margin of each lateral lobe with two concavities; visible part of tegmina ovate, apex narrowly rounded, 2.8 times as long as wide (Fig. 5A, C); hind wings developed, reaching or slightly surpassing beyond apex of hind process of pronotum; fore and mid femora flat, upper margins of all femora and lower margins of hind femora finely dentate, sawteeth of lower margins of fore and mid femora elongate, forming comb-shaped; upper margin of fore femur slightly arcuate, distal part of lower margin incomplete; upper margin of mid femur nearly straight, lower margin slightly undulate (Fig. 6F, G); width of mid femur distinctly wider than visible part of tegmen; hind femur about 2.9 times as long as wide, antegenicular triangulate, genicular denticles fingered extending backward and apex quadrangular (Fig. 5A); outer side of hind tibia with 7–8 spines, inner side with 4–5 spines; first segment of posterior tarsus equal to third in length, three pulvilli nearly equal in length, apices of all pulvilli obtuse.

*Abdomen*. Ovipositor: upper valvulae about 3.0 times as long as wide, outer margins of upper and lower valvulae with small saw-like teeth (Fig. 6E); posterior margin of subgenital plate truncate and in the middle triangularly protruding which is folded inward (Fig. 6B).

*Coloration*. Body gray. Antenna brown, color of distal segments darker; hind wings black; all femora with the same color as body; lower outer side and inner side of hind femur partially dark brown; all tibiae yellowish brown, with three black bands (the distal band longest) respectively.

#### Male.

Unknown.

#### Measurements.

Length of body (from vertex to apex of abdomen) ♀8.5–9.0 mm; length of pronotum ♀11.5–12.0 mm; length of hind femur ♀5.5–6.0 mm.

#### Type material.

Holotype female (Nos. 15-0633, MFLU), Thailand, Chiang Rai, Fathai, N20°2'58", E99°52'43", 425.5 m alt, 10 Dec. 2014, collected by Ling-Sheng ZHA. Paratypes: 2 females (HNU), same data as holotype.

#### Biology and ecology.

*Teredorus combfemorus* Zha & Hyde, sp. n. inhabits stony place on border of a stream in tropical region. From collecting time we infer they overwinter as adults.

#### Etymology.

This new species’ name is derived from the Latin *comb* and *femora*, which means lower margins of fore and mid femora comb-shaped.

#### Distribution.

Thailand (Chiang Rai).

## Discussion

Species of *Hedotettix* generally inhabit open meadow in semi-humid subtropical or tropical regions. Partial body surfaces are often covered by bright green when alive which is just like color of the meadow where they inhabit, but the green will be faded gradually after their deaths or with the change of seasons. Together with their smooth body surface, we infer that most of life cycles they live on the ground instead of in soil, and maybe most of them cannot overwinter as adults.

*Teredorus chiangraiensis* sp. n. and *Teredorus combfemorus* sp. n. are easily differed from other species of the genus by posterior angle of lateral lobe of pronotum nearly truncate (while rounded occur in all other species (Deng et al. 2014)). Based on molecular phylogeny using cytochrome c oxidase subunit I (CO I) gene (Fang et al. 2010) and morphological characteristics, Deng et al. (2014) thought *Teredorus* and *Systolederus* (Metrodorinae) can combine to the same genus, after all, they are mainly different by shape of posterior angle of lateral lobe of pronotum. Truncate posterior angle of two new species from Thailand provides a new important support for this inference, also we infer Tetriginae are not monophyly in their phylogeny and evolution.

## Supplementary Material

XML Treatment for Hedotettix

XML Treatment for Hedotettix gracilis

XML Treatment for Hedotettix triangularis

XML Treatment for Teredorus

XML Treatment for Teredorus chiangraiensis

XML Treatment for Teredorus combfemorus
